# Supplementary material for: Comparison of clinical outcomes of angiotensin receptor blockers with angiotensin-converting enzyme inhibitors in patients with acute myocardial infarction
Source: PLoS One. 2023 Sep 14;18(9):e0290251. doi: 10.1371/journal.pone.0290251 (PMC10501560; doi:10.1371/journal.pone.0290251)
Supplement: S1 Table — (PDF) [file pone.0290251.s001.pdf]

Supplementary Table 1. Disease diagnostic coding and ATC code for medication		
**Clinical outcomes or comorbidities**	**ICD-9-CM**	**ICD-10**
**AMI**	410	I2101, I2102, I2109, I2111, I2119, I2121, I2129, I213, I214, I220, I221, I222, I228, and I229
**Ischemic stroke**	433, 434, and 436	I6300, I63011, I63012, I63019, I6302, I63031, I63032, I63039, I6309, I6310, I63111, I63112, I63119, I6312, I63131, I63132, I63139, I6319, I6320, I63211, I63212, I63219, I6322, I63231, I63232, I63239, I6329, I6330, I63311, I63312, I63319, I63321, I63322, I63329, I63331, I63332, I63339, I63341, I63342, I63349, I6339, I6340, I63411, I63412, I63419, I63421, I63422, I63429, I63431, I63432, I63439, I63441, I63442, I63449, I6349, I6350, I63511, I63512, I63519, I63521, I63522, I63529, I63531, I63532, I63539, I63541, I63542, I63549, I6359, I636, I638, I639, I6501, I6502, I6503, I6509, I651, I6521, I6522, I6523, I6529, I658, I659, I6601, I6602, I6603, I6609, I6611, I6612, I6613, I6619, I6621, I6622, I6623, I6629, I663, I668, I669, and I6789
**Heart failure**	428	I501, I5020, I5021, I5022, I5023, I5030,

		I5031, I5032, I5033, I5040, I5041, I5042, I5043, and I509
**AKI**	584, 6343, 6353, 6363, 6373, 6383, 6393, 6693, and 9585	N170, N171, N172, N178, N179, O0332, O0382, O0482, O0732, O084, O904, and T795XXA
**CAD**	411.81, 411.89, 414.00, 414.01, 414.02, 414.03, 414.04, and 414	
**Hypertension**	401–405	
**Diabetes**	250	
**Hyperlipidemia**	272	
**PAOD**	440.2, 440.3, 440.8, 440.9, 443, 444.0, 444.22, 444.8, 447.8, and 447.9	
**Stroke**	430–432, 433, 434, and 436	
**Atrial fibrillation and flutter**	427.3	
**Ventricle tachycardia**	427.1	
**Ventricle fibrillation or flutter**	427.41 and 427.42	
**Gout/hyperuricemia**	274	
**intracerebral hemorrhage**	430, 431, 432, 432.0, 432.1, and 432.9	
**Gastrointestinal bleeding**	456.0, 456.20, 530.7, 530.82, 531.0, 531.2, 531.4, 531.6, 532.0, 532.2, 532.4, 532.6, 533.0, 533.2, 533.4, 533.6, 534.0, 534.2, 534.4, 534.6, 535.01, 535.11, 535.21, 535.31, 535.41, 535.51, 535.61, 537.83, 562.02, 562.03, 562.12, 562.13, 569.3, 569.85, 578.0, 578.1, and 578.9	
**Other noncritical site bleeding**	287.8, 287.9, 599.7, 596.7, 770.3, 784.7, 784.8, and 786.3	
**CKD**	585, 40301, 40311, 40391, 40402, 40403, 40412, 40413, 40492, and 40493	
**CLD**	490–496	
**Cancer**	140–208	
**Medications**	**ATC code**	
**ACEI**	C09A and C09B	
**ARB**	C09C and C09D	
**Aspirin**	B01AC06	
**Clopidogrel**	B01AC04	
**Ticagrelor**	B01AC24	
**Ticlopidine**	B01AC05	
**CCB**	C08C	
**Insulin**	A10A	
**PPIs**	A02BC	
**Warfarin**	B01AA03	
**NOACs**	B01AF02, B01AE07, B01AF01, and B01AF03	
**NSAIDs**	M01A	
**Urate-lowering agent**	M04A	
**Beta-blocker**	C07	
**Statins**	C10AA, C10BA, and C10BX	

Abbreviations: AMI = acute myocardial infarction; ACEI = angiotensin-converting enzyme inhibitor; ARB =

angiotensin receptor blocker; CAD = chronic artery disease; CCB = calcium channel blocker; CKD = chronic kidney disease; CLD = chronic lung disease; NOACs = novel oral anticoagulants; NSAIDs = nonsteroidal anti-inflammatory drugs; PAOD = peripheral arterial occlusive disease; PPIs = proton pump inhibitors; ATC = Anatomical Therapeutic Chemical; AKI = acute kidney disease; ICD-9-CM = International Classification of Diseases, Ninth Edition, Clinical Modification; ICD-10 = International Classification of Diseases, Tenth Edition
