# Supplementary material for: Comparison of clinical outcomes of angiotensin receptor blockers with angiotensin-converting enzyme inhibitors in patients with acute myocardial infarction
Source: PLoS One. 2023 Sep 14;18(9):e0290251. doi: 10.1371/journal.pone.0290251 (PMC10501560; doi:10.1371/journal.pone.0290251)
Supplement: S2 Table — (PDF) [file pone.0290251.s002.pdf]

Supplementary Table 2. Changes in the use of renin-angiotensin system inhibitors after 3 months of index date

Initial drug (after PSM)	ACEI		ARB	
**N = 11,471**				
**Switch n (%)**	8015 (53.4%)		3456 (23.1%)	
**Switch time (month)**				
**Mean, SD**	26.9	29.6	35.8	32.3
**Median (Q1–Q3)**	14.4	(6.3–36.7)	25.2	(10.7–51.1)

Abbreviations: ACEI = angiotensin-converting enzyme inhibitor; ARB = angiotensin receptor blocker; SD= standard deviation.
