# Supplementary material for: Comparison of clinical outcomes of angiotensin receptor blockers with angiotensin-converting enzyme inhibitors in patients with acute myocardial infarction
Source: PLoS One. 2023 Sep 14;18(9):e0290251. doi: 10.1371/journal.pone.0290251 (PMC10501560; doi:10.1371/journal.pone.0290251)
Supplement: S3 Table — (PDF) [file pone.0290251.s003.pdf]

**Supplementary Table 3. Incidence (per 1000 PM) and adjusted hazard risk of major outcomes for treatment efficiency based on as-treated analysis**							
**Outcomes**	Follow-up period	Treatment	Number of Events	PM	Incidence (95% CI)	Adjusted* HR (95% CI)	P
**All-cause death**	12M	ACEI	702	143,192	4.90 (4.55–5.28)	1.00 (Ref.)	
		ARB	529	168,138	3.15 (2.88–3.43)	0.65 (0.58–0.74)	<0.001
	24M	ACEI	1072	254,599	4.21 (3.96–4.47)	1.00 (Ref.)	
		ARB	940	320,513	2.93 (2.75–3.13)	0.70 (0.64–0.78)	<0.001
**MACE**	12M	ACEI	674	140,343	4.80 (4.45–5.18)	1.00 (Ref.)	
		ARB	699	165,246	4.23 (3.92–4.56)	0.87 (0.78–0.98)	0.019
	24M	ACEI	941	247,445	3.80 (3.56–4.05)	1.00 (Ref.)	
		ARB	1084	312,258	3.47 (3.27–3.68)	0.91 (0.83–1.01)	0.064
**CV death**	12M	ACEI	159	143,192	1.11 (0.94–1.30)	1.00 (Ref.)	
		ARB	118	168,138	0.70 (0.58–0.84)	0.63 (0.49–0.82)	<0.001
	24M	ACEI	214	254,599	0.84 (0.73–0.96)	1.00 (Ref.)	
		ARB	218	320,513	0.68 (0.59–0.78)	0.75 (0.61–0.93)	0.010
**Ischemic stroke**	12M	ACEI	148	142,496	1.04 (0.88–1.22)	1.00 (Ref.)	
		ARB	163	167,304	0.97 (0.83–1.14)	0.98 (0.77–1.26)	0.902
	24M	ACEI	228	252,647	0.90 (0.79–1.03)	1.00 (Ref.)	
		ARB	280	318,041	0.88 (0.78–0.99)	1.03 (0.85–1.26)	0.763
**AMI**	12M	ACEI	420	141,008	2.98 (2.70–3.28)	1.00 (Ref.)	
		ARB	460	166,021	2.77 (2.52–3.04)	0.92 (0.79–1.06)	0.241
	24M	ACEI	587	249,268	2.35 (2.17–2.55)	1.00 (Ref.)	
		ARB	672	314,594	2.14 (1.98–2.30)	0.92 (0.82–1.05)	0.206
**HHF**	12M	ACEI	1066	137,432	7.76 (7.30–8.24)	1.00 (Ref.)	
		ARB	1078	162,398	6.64 (6.25–7.05)	0.86 (0.78–0.94)	0.001
	24M	ACEI	1385	241,041	5.75 (5.45–6.06)	1.00 (Ref.)	
		ARB	1479	305,016	4.85 (4.60–5.10)	0.89 (0.82–0.96)	0.005

*Adjusted HR was estimated through stratification Cox regression adjusted for covariates listed in Table 1.

Abbreviations: AMI = acute myocardial infarction; ACEI = angiotensin-converting enzyme inhibitor; ARB = angiotensin receptor blocker; CI = confidence interval; HHF = hospitalization for heart failure; HR = hazard ratio; M = month; MACE = major adverse cardiovascular events; PM = person month; Ref. = reference; CV = cardiovascular.
