# Supplementary material for: Comparison of clinical outcomes of angiotensin receptor blockers with angiotensin-converting enzyme inhibitors in patients with acute myocardial infarction
Source: PLoS One. 2023 Sep 14;18(9):e0290251. doi: 10.1371/journal.pone.0290251 (PMC10501560; doi:10.1371/journal.pone.0290251)
Supplement: S4 Table — (PDF) [file pone.0290251.s004.pdf]

Supplementary Table 4. Incidence (per 1000 PM) and adjusted hazard risk of renal outcomes for treatment safety based on as-treated analysis

Outcomes	Follow-up period	Treatment	Number of events	PM	Incidence (95% CI)	Adjusted* HR (95% CI)	P
**AKI**	12M	ACEI	210	142,378	1.47 (1.28–1.69)	1.00 (Ref.)	
		ARB	207	167,304	1.24 (1.07–1.42)	0.79 (0.64–0.98)	0.033
	24M	ACEI	308	252,643	1.22 (1.09–1.36)	1.00 (Ref.)	
		ARB	336	318,106	1.06 (0.95–1.18)	0.82 (0.69–0.98)	0.032
**Dialysis**	12M	ACEI	54	142,953	0.38 (0.28–0.49)	1.00 (Ref.)	
		ARB	76	167,865	0.45 (0.36–0.57)	1.12 (0.77–1.62)	0.567
	24M	ACEI	85	253,929	0.33 (0.27–0.41)	1.00 (Ref.)	
		ARB	133	319,568	0.42 (0.35–0.49)	1.09 (0.80–1.49)	0.581

*Adjusted HR was estimated through stratification Cox regression adjusted for covariates listed in Table 1.

Abbreviations: AKI = acute kidney disease; ACEI = angiotensin-converting enzyme inhibitors; ARB = angiotensin receptor blocker; CI = confidence interval; HR = hazard ratio; M = month; PM = person month; Ref. = reference.
