# Supplementary material for: Comparison of clinical outcomes of angiotensin receptor blockers with angiotensin-converting enzyme inhibitors in patients with acute myocardial infarction
Source: PLoS One. 2023 Sep 14;18(9):e0290251. doi: 10.1371/journal.pone.0290251 (PMC10501560; doi:10.1371/journal.pone.0290251)
Supplement: Attachment — Submitted filename: PONE-D-23-06580.pdf [file pone.0290251.s005.pdf]

PLOS ONE

Comparison of Clinical Outcomes of Angiotensin Receptor Blockers with Angiotensin-Converting Enzyme Inhibitors in Patients with Acute Myocardial Infarction

--Manuscript Draft--

**Manuscript Number:**	PONE-D-23-06580
**Article Type:**	Research Article
**Full Title:**	Comparison of Clinical Outcomes of Angiotensin Receptor Blockers with Angiotensin-Converting Enzyme Inhibitors in Patients with Acute Myocardial Infarction
**Short Title:**	Outcomes of ARBs and ACEIs in patients with AMI
**Corresponding Author:**	Chun-Yao Huang<br>Department of Internal Medicine<br>Taipei, TAIWAN
**Keywords:**	Angiotensin receptor blockers; Angiotensin-converting enzyme inhibitors; Myocardial infarction; Mortality
**Abstract:**	Background<br>Angiotensin receptor blockers (ARBs) are considered an alternative to angiotensin-converting enzyme inhibitors (ACEIs) in patients with acute myocardial infarction (AMI), but in the era of extensive use of preventive therapies and percutaneous coronary intervention, this has not been adequately evaluated in Asians.<br>Methods<br>This retrospective cohort study used data from the Taiwan National Health Insurance Research Database. In total, 52,620 patients initially hospitalized due to AMI between 2002 and 2015 were assessed.<br>Results<br>After propensity score matching, 14,993 patients each were assigned to ACEI and ARB groups. Patients who received ARBs had significantly lower all-cause mortality (adjusted hazard ratio [aHR]: 0.82; 95% confidence interval [CI]: 0.75–0.90) and hospitalization for heart failure (aHR: 0.92; 95% CI: 0.85–0.99) compared with those who received ACEIs at 18 month follow-up. No significant difference was observed between the two groups in terms of major adverse cardiovascular events (aHR: 098; 95% CI: 0.90–1.07), cardiovascular death (aHR: 0.82; 95% CI: 0.68–1.00), ischemia stroke (aHR: 0.93; 95% CI: 0.77–1.11), and nonfatal myocardial infarction (aHR: 1.04; 95% CI: 0.93–1.17). ARBs showed benefits in many subgroups in terms of all-cause mortality and cardiovascular death.<br>Conclusions<br>Real-world data demonstrate that ARB might be associated with lower all-cause mortality and hospitalization for heart failure compared with ACEI among patients with AMI.
**Order of Authors:**	Chih-Wei Chen, M.D.
	Chun-Wei Chang
	Yi-Cheng Lin
	Wan-Ting Chen
	Li-Nien Chien
	Chun-Yao Huang
**Additional Information:**	
**Question**	**Response**
**Financial Disclosure**<br><br>Enter a financial disclosure statement that describes the sources of funding for the work included in this submission. Review	The author(s) received no specific funding for this work.

Powered by Editorial Manager® and ProduXion Manager® from Aries Systems Corporation

the submission guidelines for detailed requirements. View published research articles from *PLOS ONE* for specific examples.<br><br>This statement is required for submission and **will appear in the published article** if the submission is accepted. Please make sure it is accurate.<br><br>**Unfunded studies**<br>Enter: *The author(s) received no specific funding for this work.*<br><br>**Funded studies**<br>Enter a statement with the following details:<br>• Initials of the authors who received each award<br>• Grant numbers awarded to each author<br>• The full name of each funder<br>• URL of each funder website<br>• Did the sponsors or funders play any role in the study design, data collection and analysis, decision to publish, or preparation of the manuscript?<br>• **NO** - Include this sentence at the end of your statement: *The funders had no role in study design, data collection and analysis, decision to publish, or preparation of the manuscript.*<br>• **YES** - Specify the role(s) played.<br><br>* typeset	
**Competing Interests**<br><br>Use the instructions below to enter a competing interest statement for this submission. On behalf of all authors, disclose any competing interests that could be perceived to bias this work—acknowledging all financial support and any other relevant financial or non-financial competing interests.<br><br>This statement is required for submission and **will appear in the published article** if the submission is accepted. Please make sure it is accurate and that any funding sources listed in your Funding Information later in the submission form are also	The authors have declared that no competing interests exist.

declared in your Financial Disclosure statement.<br><br>View published research articles from *PLOS ONE* for specific examples.<br><br>**NO authors have competing interests**<br><br>Enter: *The authors have declared that no competing interests exist.*<br><br>**Authors with competing interests**<br><br>Enter competing interest details beginning with this statement:<br><br>*I have read the journal's policy and the authors of this manuscript have the following competing interests: [insert competing interests here]*<br><br>* typeset	
**Ethics Statement**<br><br>Enter an ethics statement for this submission. This statement is required if the study involved:<br><br>• Human participants<br>• Human specimens or tissue<br>• Vertebrate animals or cephalopods<br>• Vertebrate embryos or tissues<br>• Field research<br><br>Write "N/A" if the submission does not require an ethics statement.<br><br>General guidance is provided below. Consult the submission guidelines for detailed instructions. **Make sure that all information entered here is included in the Methods section of the manuscript.**	This study was approved by the Joint Institutional Review Board of Taipei Medical University (TMU-JIRB No. 201911004). The need for informed consent was waived owing to the use of anonymized data.

**Format for specific study types**<br><br>**Human Subject Research (involving human participants and/or tissue)**<br>• Give the name of the institutional review board or ethics committee that approved the study<br>• Include the approval number and/or a statement indicating approval of this research<br>• Indicate the form of consent obtained (written/oral) or the reason that consent was not obtained (e.g. the data were analyzed anonymously)<br><br>**Animal Research (involving vertebrate animals, embryos or tissues)**<br>• Provide the name of the Institutional Animal Care and Use Committee (IACUC) or other relevant ethics board that reviewed the study protocol, and indicate whether they approved this research or granted a formal waiver of ethical approval<br>• Include an approval number if one was obtained<br>• If the study involved *non-human primates*, add *additional details* about animal welfare and steps taken to ameliorate suffering<br>• If anesthesia, euthanasia, or any kind of animal sacrifice is part of the study, include briefly which substances and/or methods were applied<br><br>**Field Research**<br><br>Include the following details if this study involves the collection of plant, animal, or other materials from a natural setting:<br>• Field permit number<br>• Name of the institution or relevant body that granted permission	
**Data Availability**<br><br>Authors are required to make all data underlying the findings described fully available, without restriction, and from the time of publication. PLOS allows rare exceptions to address legal and ethical concerns. See the PLOS Data Policy and FAQ for detailed information.	Yes - all data are fully available without restriction

A Data Availability Statement describing where the data can be found is required at submission. Your answers to this question constitute the Data Availability Statement and **will be published in the article**, if accepted.<br><br>**Important:** Stating 'data available on request from the author' is not sufficient. If your data are only available upon request, select 'No' for the first question and explain your exceptional situation in the text box.<br><br>Do the authors confirm that all data underlying the findings described in their manuscript are fully available without restriction?	
**Describe where the data may be found in full sentences. If you are copying our sample text, replace any instances of XXX with the appropriate details.**<br><br>• If the data are **held or will be held in a public repository**, include URLs, accession numbers or DOIs. If this information will only be available after acceptance, indicate this by ticking the box below. For example: *All XXX files are available from the XXX database (accession number(s) XXX, XXX.)*.<br>• If the data are all contained **within the manuscript and/or Supporting Information files**, enter the following: *All relevant data are within the manuscript and its Supporting Information files.*<br>• If neither of these applies but you are able to provide **details of access elsewhere**, with or without limitations, please do so. For example:<br><br>*Data cannot be shared publicly because of [XXX]. Data are available from the XXX Institutional Data Access / Ethics Committee (contact via XXX) for researchers who meet the criteria for access to confidential data.*<br><br>*The data underlying the results presented in the study are available from (include the name of the third party*	All relevant data are within the manuscript and its Supporting Information files.

and contact information or URL).<br>• This text is appropriate if the data are owned by a third party and authors do not have permission to share the data.<br><br>* typeset	
Additional data availability information:	Tick here if the URLs/accession numbers/DOIs will be available only after acceptance of the manuscript for publication so that we can ensure their inclusion before publication.

Powered by Editorial Manager® and ProduXion Manager® from Aries Systems Corporation

Manuscript

Click here to access/download;Manuscript;Manuscript 2023 (final 0328 re3).docx

Comparison of Clinical Outcomes of Angiotensin Receptor Blockers with Angiotensin-Converting Enzyme Inhibitors in Patients with Acute Myocardial Infarction

Chih-Wei Chen, MD[a,b,c,1]; Chun-Wei Chang MD[a,b,1] ;Yi-Cheng Lin, PHARM D[d,e]; Wan-Ting Chen[f]; Li-Nien Chien, PHD[*f,g]; Chun-Yao Huang, PHD[*a,b,c]

[a] Division of Cardiology, Department of Internal Medicine and Cardiovascular Research Center, Taipei Medical University Hospital, Taipei, Taiwan

[b] Taipei Heart institute, Taipei Medical University, Taipei, Taiwan

[c] Division of Cardiology, Department of Internal Medicine, School of Medicine, College of Medicine, Taipei Medical University, Taipei, Taiwan

[d] Department of Pharmacy, Taipei Medical University Hospital, Taipei, Taiwan

[e] School of Pharmacy, College of Pharmacy, Taipei Medical University, Taipei, Taiwan

[f] Institute of Health and Welfare Policy, College of Medicine, National Yang Ming Chiao Tung University, Taipei, Taiwan

[g] Graduate Institute of Data Science, College of Management, Taipei Medical University, Taipei, Taiwan

1 first author

Chih-Wei Chen and Chun-Wei Chang contributed equally to this work

* Corresponding author

Li-Nien Chien and Chun-Yao Huang contributed equally to this work

Short title: Outcomes of ARBs and ACEIs in patients with AMI

Word count of the abstract: 217

Word count of the text: 2784 (NOT including abstract, tables, figures, acknowledgments, funding, references)

Number of tables: 3

Number of figures: 3

Abstract

Background

Angiotensin receptor blockers (ARBs) are considered an alternative to angiotensin-converting enzyme inhibitors (ACEIs) in patients with acute myocardial infarction (AMI), but in the era of extensive use of preventive therapies and percutaneous coronary intervention, this has not been adequately evaluated in Asians.

Methods

This retrospective cohort study used data from the Taiwan National Health Insurance Research Database. In total, 52,620 patients initially hospitalized due to AMI between 2002 and 2015 were assessed.

Results

After propensity score matching, 14,993 patients each were assigned to ACEI and ARB groups. Patients who received ARBs had significantly lower all-cause mortality (adjusted hazard ratio [aHR]: 0.82; 95% confidence interval [CI]: 0.75–0.90) and hospitalization for heart failure (aHR: 0.92; 95% CI: 0.85–0.99) compared with those who received ACEIs at 18 month follow-up. No significant difference was observed between the two groups in terms of major adverse cardiovascular events (aHR: 098; 95% CI: 0.90–1.07), cardiovascular death (aHR: 0.82; 95% CI: 0.68–1.00), ischemia stroke (aHR: 0.93; 95% CI: 0.77–1.11), and nonfatal myocardial infarction (aHR: 1.04; 95% CI: 0.93–1.17). ARBs showed benefits in many subgroups in terms of all-cause mortality and

cardiovascular death.

Conclusions

Real-world data demonstrate that ARBs might be associated with lower all-cause mortality and hospitalization for heart failure compared with ACEIs among patients with AMI.

Keywords

Angiotensin receptor blockers; Angiotensin-converting enzyme inhibitors; Myocardial infarction; Mortality

Introduction

Angiotensin-converting enzyme inhibitors (ACEIs) and angiotensin receptor blockers (ARBs) have been recommended as preventive therapies for patients with acute myocardial infarction (AMI) in the European Society of Cardiology and American College of Cardiology/American Heart Association (ACC/AHA) guidelines[1-3]. ACEIs were recommended as standard treatment in patients with myocardial infarction (MI), particularly in patients with left ventricular systolic dysfunction, diabetes, or chronic kidney disease. By contrast, ARBs are considered alternatives to ACEIs when patients are intolerant to ACEIs[1-3]. The prescription rate of ARBs has prominently increased worldwide over the past several years[4, 5]. This phenomenon is significant in Asian countries because persistent cough is a common side effect associated with ACEI therapy among Eastern Asians[6, 7]. Many trials have demonstrated the beneficial roles of ACEI in patients with

AMI [8, 9], and ARBs have been found to be noninferior to ACEIs in patients with AMI for major adverse cardiovascular event (MACE) outcomes[10, 11]. However, ARBs have not been adequately compared with ACEIs in Asians with AMI, particularly in the modern era with extensive use of statins and timely percutaneous coronary intervention (PCI). Additionally, the use of ACEI or ARB could result in acute kidney injury (AKI) and hyperkalemia; thus, adverse events are common in patients with chronic kidney disease (CKD) [12, 13]. Furthermore, evidence of renal outcomes after ACEI/ARB treatment of patients with AMI, followed by multiple comorbidities, is rare. Therefore, we conducted this study to investigate the effectiveness of ACEI and ARB in terms of mortality, MACE, hospitalization for heart failure (HHF), and renal outcomes in Asians with AMI by using the data from real-world settings.

Material and methods

Ethics statement

This study was approved by the Joint Institutional Review Board of Taipei Medical University (TMU-JIRB No. 201911004). The need for informed consent was waived owing to the use of anonymized data.

Study design and data source

This study adopted a retrospective cohort design and used data files from National Health Insurance Research Database (NHIRD) in Taiwan. NHIRD is a population-based claims database of the

National Health Insurance (NHI) program, a single-payer health insurance program initiated in 1995. NHI provides a comprehensive coverage in Taiwan, including outpatient visits, the inpatient system, drug prescriptions, treatment with traditional Chinese medicine, dental services, operations, and examinations through radiographic and magnetic resonance images. Under the legislation, all legal residents of Taiwan are eligible for NHI benefit and are required to enroll for the program; therefore, the coverage of NHI reached 99.9% of the Taiwanese population by the end of 2017. Vital status and cause of death were ascertained from the National Death Registry of Taiwan. The two datasets can be linked by a unique encrypted identifier under the regulation of Health and Welfare Data Science Center, Ministry of Health and Welfare, Taiwan.

Study cohort

Patients hospitalized with a diagnosis of AMI between 2002 and 2015 were first selected, and the date of first AMI admission was treated as the index date of AMI. Those patients' data was first accessed on December 1st, 2019. To confirm the diagnosis, only patients who received heparin or antiplatelet agents during their AMI admission were included in the study. We excluded patients (1) aged <20 years, (2) with missing sex information, (3) who were not a citizen of Taiwan, (4) who had undergone coronary artery bypass or had a history of AMI (a 2-year washout period), (5) had a death record or AMI or stroke hospitalization within 3 months after the index date of AMI, and (6) had a dialysis claims before or within 3 months after the index date of AMI. Moreover, we excluded

patients who were hospitalized for >1 month because it indicated that their disease condition was complicated.

. Therefore, we additionally excluded patients who did not receive ACEI or ARB or received both ACEI and ARB or switched one medication to another or had a medication possession ratio (MPR) of <0.4 within 3 months after the index date of AMI. The MPR was determined based on the proportion of days on medication within 3 months. Finally, the patients were classified into the ACEI or ARB group within 3 months after the index date of AMI. The detailed flow chart of patient selection is presented in **Fig. 1**.

Fig. 1. Patient selection process.
Abbreviations: AMI = acute myocardial infarction; ACEI = angiotensin-converting enzyme inhibitor; ARB = angiotensin receptor blocker; CABG = coronary artery bypass; MPR = medication possession ratio.

Propensity score matching

To reduce the potential selection bias, we used one-to-one propensity score matching (PSM) to select a pair of patients who had similar baseline characteristics but different treatments (either ACEI or ARB), so that the two groups could have similar distributions (to a comparable degree) of observed baseline covariates. We applied the nearest neighbor matching within 0.1 caliper distance of propensity score to select patients and considered the covariates of age, sex, diagnostic year, comorbidities, and medication use to estimate the propensity score (Table 1). A logistic regression analysis was used to calculate propensity scores. The similarity between the two groups was evaluated through standardized mean difference (SMD), and a SMD of <10% (or 0.1) indicated a

negligible correlation between the treatment groups and variables.

The underlying comorbidities of participants included vascular comorbidities such as coronary artery disease (CAD), hypertension, diabetes, hyperlipidemia, peripheral arterial occlusive disease, heart failure, stroke, atrial fibrillation, and ventricle diseases; kidney diseases such as hyperuricemia and chronic kidney disease; and a history of bleeding. Medication use both before and after AMI were considered, including antiplatelet agents, clopidogrel, ACEI/ARB, statin, acetylsalicylic acid/dipyridamole, diuretics, calcium channel blockers, and beta-blockers. The International Classification of Diseases (ICD), Ninth Edition, Clinical Modification and ICD, Tenth Edition for disease diagnostic codes and the Anatomical Therapeutic Chemical code for medications are listed in Appendix 1. Finally, PCI, IAPD, and extracorporeal membrane oxygenation use before the index date of AMI were considered to adjust the baseline difference between the two groups.

Main outcome measurements

Outcomes of treatment effectiveness were all-cause mortality, MACE, and HHF. MACE was defined as cardiovascular (CV) death, nonfatal myocardial infraction, and nonfatal ischemic stroke during the follow-up periods. Death records were derived from the National Death Registry, which provided the death date and death cause. Nonfatal myocardial infraction, stroke, and HHF were derived based on discharge records from NHIRD. Safety outcomes were AKI and renal failure. AKI was defined as a

discharge diagnosis of AKI in conjunction with a procedure code of dialysis. Dialysis was defined as receiving dialysis continuously for 3 months. (**Supplementary Table 1**)

Statistical analysis

Descriptive statistics was used to present the characteristics of the ACEI and ARB groups. This study was mainly designed as an intention-to-treat analysis that ignores noncompliance and drug switching or drug withdrawal after enrollment. We believe that this approach can provide a good estimate of treatment efficiency when patients with a high adherence level are included. Additionally, we provided an as-treated analysis as a sensitivity analysis, in which the data of patients were censored if they switched or discontinued their initial medications.

Survival analysis along with Kaplan–Meier method and a stratified Cox proportional hazard regression was used to compare the outcome risk between ACEI and ARB. The follow-up periods were set at 12 and 18 months. Patients were followed from 3 months after the index date of AMI to whichever outcome occurred first: (1) the outcome date; (2) the death date; (3) end of the observation period; or (4) December 31, 2017. The cumulative event rates of interest were estimated using the Kaplan–Meier method. Furthermore, the incidence of events along with the 95% confidence interval (CI) was computed. A stratified Cox proportional hazard regression was used to compare the event risk between ACEI and ARB. The assumption of proportional hazards was assessed. All analyses were performed using SAS/STAT 9.4 software (SAS Institute Inc., Cary, NC, USA), STATA 14 software (Stata Corp LP, College Station, TX, USA), and R (version 3.2.5 for Windows). A value of

P < 0.05 was considered significant.

Results

Baseline patient characteristics

We identified 52,620 patients who survived AMI after receiving either ACEIs or ARBs between January 2002 and December 2015. After PSM, 14,993 patients each were assigned to ACEI and ARB arms. As shown in **Table 1**, the rate of ARB prescription increased from 13.3% in 2002–2005 to 54.1% in 2011–2015 among patients with AMI. By contrast, the prescription rate of ACEIs increased slightly from 26.7% in 2002–2005 to 35.2% in 2011–2015. Before PSM, patients receiving ARBs were older and more likely to have comorbidities such as CAD, hypertension, diabetes mellitus, heart failure, and chronic kidney disease than those receiving ACEI. However, these intergroup differences in baseline characteristics were well-balanced after PSM, with the SMDs being <0.1 for all characteristics. The detailed baseline characteristics, underlying comorbidities, medication histories, and in-hospital treatment characteristics are presented in **Table 1**.

Table 1. Baseline characteristics of new AMI survivors who received either ACEI or ARB within 3 months after discharge

	Before PSM			After PSM		
	ACEI	ARB		ACEI	ARB	
	(%)	(%)	SMD	(%)	(%)	SMD
**Sample size, n**	27,329	25,291		14,993	14,993	
**Male**	82.1	71.3	0.256	81.5	81.5	<0.001

Age, years						
Mean (SD)	61.83 (13.85)	65.36 (13.66)	0.257	62.92 (13.13)	62.92 (13.14)	0.001
20–44	10.6	7.0	0.128	8.2	8.1	0.002
45–64	47.6	40.0	0.152	46.8	46.8	<0.001
65–74	20.5	23.6	0.074	22.7	22.5	0.004
≥75	21.4	29.4	0.186	22.3	22.5	0.005
DX year						
2002–2005	26.7	13.3	0.339	16.4	16.4	<0.001
2006–2010	38.2	32.7	0.116	35.4	35.4	<0.001
2011–2015	35.2	54.1	0.387	48.2	48.2	<0.001
STEMI	74.9	60.3	0.315	71.0	66.6	0.095
History of cardiac procedure, yes						
Prior PCI	1.3	2.7	0.107	1.6	1.7	0.006
IABP	3.6	3.8	0.014	4.0	4.0	0.003
ECMO	0.2	0.2	0.015	0.2	0.2	0.003
Comorbidities, yes						
CAD	67.2	73.9	0.149	74.8	72.6	0.050
Hypertension	62.8	79.0	0.362	69.5	70.3	0.016
Diabetes	33.3	41.1	0.163	35.0	34.5	0.010
Hyperlipidemia	51.7	52.8	0.023	53.3	52.5	0.016
PAOD	2.4	3.3	0.054	2.6	2.7	0.006
Heart failure	44.7	51.7	0.139	46.4	47.2	0.016
Stroke	6.8	8.4	0.062	6.8	6.6	0.008
Atrial fibrillation or flutter	5.8	7.9	0.085	6.1	6.1	0.001
Ventricle tachycardia	2.3	2.1	0.012	2.3	2.3	0.004
Ventricular fibrillation or flutter	1.5	1.3	0.016	1.4	1.5	0.007
Gout or hyperuricemia	11.5	12.0	0.013	11.0	12.1	0.033
Intracerebral hemorrhage	0.6	0.7	0.012	0.6	0.6	0.003
Gastrointestinal bleeding	5.1	6.0	0.039	5.0	4.9	0.004
Other noncritical site bleeding	1.7	2.1	0.029	1.9	1.9	0.001
CKD	3.8	7.7	0.172	4.4	4.3	0.004
CLD	11.0	15.0	0.119	11.6	12.6	0.031
Cancer	3.0	4.1	0.059	3.6	3.4	0.010
Medication use, yes						
Aspirin	17.1	25.0	0.193	17.9	17.9	0.001
Clopidogrel	2.0	4.2	0.128	2.2	2.2	0.005

Ticlopidine	0.9	1.2	0.028	0.9	0.8	0.013
CCB	23.2	35.7	0.277	26.5	26.8	0.008
Insulin	2.8	5.7	0.146	3.1	3.2	0.005
PPIs	2.4	3.3	0.053	2.6	2.5	0.005
Warfarin	0.8	1.2	0.038	0.8	0.8	<0.001
NOACs	0.0	0.1	0.030	0.0	0.0	0.010
NSAIDs	35.1	38.2	0.065	35.4	35.8	0.008
Urate-lowering agent	8.7	11.1	0.083	8.7	9.4	0.024
ACEI	14.6	8.5	0.192	10.5	10.0	0.017
ARB	10.9	38.2	0.667	15.9	16.5	0.017
Beta-blocker	18.7	28.4	0.229	20.4	20.7	0.007
Statins	11.3	19.7	0.234	13.2	12.8	0.015
**Medication use within 3 months after the index AMI discharge**						
Aspirin	91.8	88.9	0.098	91.8	91.4	0.014
Clopidogrel	77.8	77.5	0.008	79.2	78.9	0.007
Ticagrelor	6.7	10.5	0.133	10.5	8.8	0.058
Ticlopidine	2.6	1.6	0.069	1.4	1.7	0.019
Beta-blocker	70.1	69.7	0.008	70.9	70.1	0.017
Statins	62.4	66.3	0.082	68.1	65.7	0.052
MPR within 90 days						
≥0.80	60.0	57.3	0.054	56.3	59.0	0.055
0.4–0.8	40.0	42.7	0.054	43.7	41.0	0.055

Abbreviation: AMI = acute myocardial infarction; ACEI = angiotensin-converting enzyme inhibitors; ARB = angiotensin receptor blocker; CAD = chronic artery disease; CCB = calcium channel blocker; CKD = chronic kidney disease; CLD = chronic lung disease; ECMO = extracorporeal membrane oxygenation; IABP = intra-aortic balloon pump; MPR = medication possession ratio; NOACs= novel oral anticoagulants; NSAIDs = nonsteroidal anti-inflammatory drugs; PAOD = peripheral arterial occlusive disease; PCI = percutaneous coronary intervention; PPIs = proton pump inhibitors; PSM = propensity score matching; SMD = standardized mean difference; SD = standard deviation. STEMI= ST segment elevation myocardial infarction.

Adherence of treatment group

Adherence to therapy was defined as a MPR of >40% within 3 months after the index date. As a result, 60.0% and 57.3% of ACEI and ARBs users, respectively, had a MPR of >0.8 within 90 days

after the index date of AMI. During follow up, 8015 (53.4%) ACEI users switched to ARBs, and the median time of switching was 14.4 (6.3–36.7) months. By contrast, 3456 (23.1%) ARBs users switched to ACEIs during this period, and the median time of switching was 25.2 (10.1–51.1) months (**Supplementary Table 2**).

Clinical outcomes

During the follow-up period, 807 and 641 ACEI and ARB users, respectively, died. For all-cause mortality at 18-month follow-up, the ARB group had significantly lower all-cause mortality than the ACEI group (adjusted hazard ratio [aHR]: 0.82; 95% CI: 0.75–0.90; **Table 2**). Kaplan–Meier survival estimates for 18-month all-cause mortality in matched cohorts are presented in **Fig. 2A**. The trend of all-cause mortality after AMI decreased from 2002 to 2015, which is demonstrated according to the year of AMI diagnosis in **Fig. 2B**. No significant difference was observed between the ARB and ACEI groups in terms of MACE (aHR: 0.98; 95% CI: 0.90–1.07), CV death (aHR: 0.82; 95% CI: 0.68–1.00), ischemia stroke (aHR: 0.93; 95% CI: 0.77–1.11), and non-fatal MI (aHR: 1.04; 95% CI: 0.93–1.17) at 18-month follow-up. However, HHF in the ARB group were significantly lower than in the ACEI groups (aHR: 0.92; 95% CI: 0.85–0.99).

For renal outcomes, no significant difference was observed between the two groups in terms of AKI (aHR: 0.93; 95% CI: 0.79–1.09) and dialysis (aHR: 1.14; 95% CI: 0.86–1.50) at 18-month follow-up (**Table 3**). The subgroups did not show heterogeneity in the effect of treatment on the risk

of 18-month mortality and CV death (**Fig. 3**).

We also performed a sensitivity analysis in which the data of patients were censored if they switched the initial medication. The results were consistent with the main findings, as shown in **Supplementary Tables 3** and **4**.

Table 2. Incidence (per 1000 PM) and adjusted hazard risk of the major outcomes for treatment efficiency based on intention-to-treat analysis

Outcomes	Follow-up period	Treatment	Number of events	PM	Incidence (95% CI)	Adjusted* HR (95% CI)	P
**All-cause death**	12M	ACEI	807	174,689	4.62 (4.31–4.95)	1.00 (Ref.)	
		ARB	641	175,783	3.65 (3.37–3.94)	0.79 (0.71–0.87)	<0.001
	18M	ACEI	1118	258,867	4.32 (4.07–4.58)	1.00 (Ref.)	
		ARB	915	261,068	3.5 (3.28–3.74)	0.82 (0.75–0.90)	<0.001
**MACE**	12M	ACEI	798	170,586	4.68 (4.36–5.01)	1.00 (Ref.)	
		ARB	764	171,827	4.45 (4.14–4.77)	0.95 (0.86–1.05)	0.334
	18M	ACEI	1020	251,221	4.06 (3.81–4.32)	1.00 (Ref.)	
		ARB	993	253,430	3.92 (3.68–4.17)	0.98 (0.90–1.07)	0.677
**CV death**	12M	ACEI	188	174,689	1.08 (0.93–1.24)	1.00 (Ref.)	
		ARB	148	175,783	0.84 (0.71–0.99)	0.76 (0.61–0.95)	0.016
	18M	ACEI	240	258,867	0.93 (0.81–1.05)	1.00 (Ref.)	
		ARB	203	261,068	0.78 (0.67–0.89)	0.82 (0.68–1.00)	0.05
**Ischemic stroke**	12M	ACE-I	182	173,646	1.05 (0.90–1.21)	1.00 (Ref.)	
		ARB	178	174,705	1.02 (0.87–1.18)	0.92 (0.74–1.14)	0.448
	18M	ACEI	259	256,809	1.01 (0.89–1.14)	1.00 (Ref.)	
		ARB	249	258,924	0.96 (0.85–1.09)	0.93 (0.77–1.11)	0.408
**AMI**	12M	ACEI	498	171,544	2.90 (2.65–3.17)	1.00 (Ref.)	
		ARB	501	172,811	2.90 (2.65–3.16)	1.02 (0.90–1.16)	0.722
	18M	ACEI	618	253,106	2.44 (2.25–2.64)	1.00 (Ref.)	

		ARB	625	255,388	2.45 (2.26–2.65)	1.04 (0.93–1.17)	0.501
**HHF**	12M	ACEI	1273	166,756	7.63 (7.22–8.07)	1.00 (Ref.)	
		ARB	1174	168,448	6.97 (6.58–7.38)	0.90 (0.82–0.97)	0.009
	18M	ACEI	1530	244,556	6.26 (5.95–6.58)	1.00 (Ref.)	
		ARB	1436	247,420	5.8 (5.51–6.11)	0.92 (0.85–0.99)	0.026

*Adjusted HR was estimated through stratification Cox regression adjusted for the covariates listed in Table 1.

Abbreviations: AMI = acute myocardial infarction; ACEI = angiotensin-converting enzyme inhibitor; ARB = angiotensin receptor blocker; CI = confidence interval; HHF = hospitalization for heart failure; HR = hazard ratio; M = month; MACE = major adverse cardiovascular events; PM = person month; Ref. = reference; CV = cardiovascular.

Table 3. Incidence (per 1000 PM) and adjusted hazard risk of renal outcomes for treatment safety based on intention-to-treat analysis							
**Outcomes**	Follows-up period	Treatment	Number of Events	PM	Incidence (95% CI)	Adjusted* HR (95% CI)	P
**AKI**	12M	ACEI	255	173,576	1.47 (1.29–1.66)	1.00 (Ref.)	
		ARB	238	174,724	1.36 (1.19–1.55)	0.93 (0.77–1.11)	0.408
	18M	ACEI	**339**	**256,788**	**1.32 (1.18–1.47)**	1.00 (Ref.)	
		ARB	317	259,035	1.22 (1.09–1.37)	0.93 (0.79–1.09)	0.355
**Dialysis**	12M	ACEI	68	174,361	0.39 (0.30–0.49)	1.00 (Ref.)	
		ARB	89	175,407	0.51 (0.41–0.62)	1.19 (0.86–1.65)	0.284
	18M	ACEI	**98**	**258,207**	**0.38 (0.31–0.46)**	1.00 (Ref.)	
		ARB	119	260,248	0.46 (0.38–0.55)	1.14 (0.86–1.50)	0.36

*Adjusted HR was estimated through stratification Cox regression adjusted for the covariates listed in Table 1.

Abbreviations: AKI = acute kidney disease; ACEI = angiotensin-converting enzyme inhibitor; ARB = angiotensin receptor blocker; CI = confidence interval; HR = hazard ratio; M = month; PM = person month; Ref. = reference.

Fig. 2. Kaplan–Meier failure curve for all-cause death based on (A) treatment and (B) treatment and diagnostic year. Abbreviations: ACEI = angiotensin-converting enzyme inhibitors; ARB = angiotensin receptor blocker.

Fig. 3. Subgroup analysis of the hazard risks of all-cause cause, CV death, and HHF within an 18-month follow-up period based on intention-to-treat analysis.
Abbreviations: AMI = acute myocardial infarction; ACEI = angiotensin-converting enzyme inhibitor; ARB = angiotensin receptor blocker; CV = cardiovascular; HHF = hospitalization for heart failure; HR = hazard ratio; M = month; PM = person month; Ref. = reference; CKD = chronic kidney disease.

Discussion

In this nationwide cohort study of survivors of AMI, it was found that the prescription rate of ARBs increased from 2002 to 2015. Compared to patients on ACEIs, those on ARBs exhibited lower rates of all-cause mortality, cardiovascular death, and HHF. However, no significant differences were observed in adverse renal outcomes between the ACEI and ARB groups..

From 2002 to 2015, the prescription rate of ARBs increased. We observed that ARBs were preferred for RAS blockade in patients with AMI, particularly in patients with high-risk features, such as heart failure. Furthermore, our study demonstrated high tolerance to ARBs and prominent drug switches of initial ACEI users after a 3-month censor period (Supplementary Table 2).

Despite the debate of the ARB-MI paradox [14], our study found that ARB users showed lower all-cause mortality than ACEI users, which is different from the results of many clinical trials. It is broadly believed that ACEIs are more effective than ARBs in reducing CV outcomes, particularly in patients with AMI [14]. Most of the placebo control trials of ACEIs have shown significantly lower mortality and more favorable CV outcomes [15], which was not observed in the placebo control trials of ARBs[16]. One possible explanation is the “generation gap” between ACEI and ARB trials. ACEI trials were conducted in an era that did not widely use secondary prevention therapies and timely PCI, which may be the cause of the higher placebo event rate than in ARBs trials. Additionally, most head-to-head comparison trials have shown no significant difference between ACEI and ARB users [10, 11]. For example, in the OPTIMAAL trial, losartan use was associated

with no significant difference in all-cause mortality but significantly higher CV death compared with captopril use [17], although some experts explained that the result could be related to the low dose of losartan used [10]. Additionally, the VALIANT trial showed no significant outcome difference between ACEI and ARB users [11]. Furthermore, results from two meta-analyses have yielded inconsistent findings, but these analyses did not focus on patients with acute myocardial infarction. [18, 19]; however, they both suggested ARBs to be as efficacious and safe as ACEIs, with the added advantage of higher tolerability when the study was restricted the trials after 2000. Most of these studies were conducted in Western countries, and few Asians were enrolled in these trials. Moreover, studies conducted in Asia have shown inconsistent results [7, 20-22]. Because clinical trials, meta-analysis, and studies using real-world data have concluded that ARBs are noninferior to ACEIs, the treatment strategy could be different in Asian countries because Asian patients usually develop adverse side effects such as cough with ACEI therapy [23].

In our study, the incidence of AKI and renal replacement therapy in ACEI and ARB users was similar at 12- and 18-month follow-up periods. Although, to the best of our knowledge, no study has compared AKI risk in patients with AMI on ACEI and ARB therapies, one study based on registry data found that ACEI/ARB therapy may resulted in an increased risk of AKI, particularly in patients with an eGFR of >30 mL/min/1.73 m^2 compared with patients without ACEI/ARB therapy [24]. The incidence of AKI and end-stage renal failure requiring dialysis in a study was 1.22–1.47 and 0.39–0.51 per 1000 person-years, respectively, which were comparable with the normal population in

Taiwan (0.43 per 1000 person-years in 2012) [25]. Although, a concern exists that renin angiotensin system inhibitor may cause adverse renal outcomes [26], particularly in CKD patients, our study showed no significant difference between ACEI and ARB users in terms of AKI and dialysis.

Our study varies from previous studies in several aspects. First, only few studies have focused on Asians. The Korean AMI registry investigated the clinical outcomes of ACEI and ARB users; however, it evaluated patients' medications only at discharge [7, 20]. Conversely, our study evaluated medication within 3 months after AMI discharge. Second, the results of the current studies were compared with those involving selected patient registries or clinical trial volunteers, although our data were derived from the daily clinical practice, which were closer to real-world data. The analysis based on generation was performed (2002–2005, 2006–2010, and 2011–2015), including bear metal stents and first- and second-generation drug eluting stents to increase the internal validity of the current study. Moreover, this study adopted several statistical tools to increase the robustness of the findings, including using PSM to reduce the selection bias, performing subgroup analysis to validate results across different groups, and conducting sensitivity analysis to ensure the medication switch did not affect the findings.

This current study has few limitations. First, the observational study design may introduce residual confounding bias, although we used the PSM to balance the major observable baseline covariates. Second, no information on individuals' exact blood pressure levels or reasons for the selection and dosage of a specific antihypertensive medication is recorded in the NHIRD.

Furthermore, we lacked information on patient characteristics such as obesity, alcohol consumption, smoking, and cognitive or functional status, left ventricular ejection fraction, and biochemistry data. Third, we used the prescription to estimate adherence, but we had no information regarding patient's actual drug adherence. However, this measure of adherence correlated with pill counts in other studies [27]. Additionally, drug interruption or switching may have led to overestimation of the effects of the respective drugs in IIT analysis.

Our study may provide clinical implications of Asians after MI. These real-world data demonstrate that AMI patients might benefit from ARBs in lowering all-cause mortality and reducing hospitalization rate for heart failure compared with ACEIs.

Acknowledgment

The authors wish to thank Wallace Academic Editing for editing this manuscript.

Declaration of Competing Interest

The authors have not declared a specific grant for this research from any funding agency in the public, commercial or not-for-profit sectors.

Reference:

1. O'Gara PT, Kushner FG, Ascheim DD, Casey DE, Jr., Chung MK, de Lemos JA, et al. 2013 ACCF/AHA guideline for the management of ST-elevation myocardial infarction: executive summary: a report of the American College of Cardiology Foundation/American Heart Association Task Force on Practice Guidelines: developed in collaboration with the American College of Emergency Physicians and Society for Cardiovascular Angiography and Interventions. Catheter Cardiovasc

Interv. 2013;82(1):E1-27. doi: 10.1002/ccd.24776. PubMed PMID: 23299937.
2. Ibanez B, James S, Agewall S, Antunes MJ, Bucciarelli-Ducci C, Bueno H, et al. 2017 ESC Guidelines for the management of acute myocardial infarction in patients presenting with ST-segment elevation: The Task Force for the management of acute myocardial infarction in patients presenting with ST-segment elevation of the European Society of Cardiology (ESC). Eur Heart J. 2018;39(2):119-77. doi: 10.1093/eurheartj/ehx393. PubMed PMID: 28886621.
3. Collet JP, Thiele H, Barbato E, Barthelemy O, Bauersachs J, Bhatt DL, et al. 2020 ESC Guidelines for the management of acute coronary syndromes in patients presenting without persistent ST-segment elevation. Rev Esp Cardiol (Engl Ed). 2021;74(6):544. doi: 10.1016/j.rec.2021.05.002. PubMed PMID: 34020768.
4. Jarari N, Rao N, Peela JR, Ellafi KA, Shakila S, Said AR, et al. A review on prescribing patterns of antihypertensive drugs. Clin Hypertens. 2015;22:7. doi: 10.1186/s40885-016-0042-0. PubMed PMID: 27019747; PubMed Central PMCID: PMCPMC4808570.
5. Komajda M, Cowie MR, Tavazzi L, Ponikowski P, Anker SD, Filippatos GS, et al. Physicians' guideline adherence is associated with better prognosis in outpatients with heart failure with reduced ejection fraction: the QUALIFY international registry. Eur J Heart Fail. 2017;19(11):1414-23. doi: 10.1002/ejhf.887. PubMed PMID: 28463464.
6. Liu J, Masoudi FA, Spertus JA, Wang Q, Murugiah K, Spatz ES, et al. Patterns of use of angiotensin-converting enzyme inhibitors/angiotensin receptor blockers among patients with acute myocardial infarction in China from 2001 to 2011: China PEACE-Retrospective AMI Study. J Am Heart Assoc. 2015;4(2). doi: 10.1161/JAHA.114.001343. PubMed PMID: 25713293; PubMed Central PMCID: PMCPMC4345866.
7. Lee JH, Bae MH, Yang DH, Park HS, Cho Y, Lee WK, et al. Angiotensin II type 1 receptor blockers as a first choice in patients with acute myocardial infarction. Korean J Intern Med. 2016;31(2):267-76. doi: 10.3904/kjim.2014.268. PubMed PMID: 26701233; PubMed Central PMCID: PMCPMC4773713.
8. GISSI-3: effects of lisinopril and transdermal glyceryl trinitrate singly and together on 6-week mortality and ventricular function after acute myocardial infarction. Gruppo Italiano per lo Studio della Sopravvivenza nell'infarto Miocardico. Lancet. 1994;343(8906):1115-22. PubMed PMID: 7910229.
9. ISIS-4: a randomised factorial trial assessing early oral captopril, oral mononitrate, and intravenous magnesium sulphate in 58,050 patients with suspected acute myocardial infarction. ISIS-4 (Fourth International Study of Infarct Survival) Collaborative Group. Lancet. 1995;345(8951):669-85. PubMed PMID: 7661937.
10. Gayet JL, Optimal Trial in Myocardial Infarction with the Angiotensin IIAL. The OPTIMAAL trial: losartan or captopril after acute myocardial infarction. Lancet. 2002;360(9348):1884-5; author reply 5. doi: 10.1016/s0140-6736(02)11745-4. PubMed PMID: 12480392.
11. Pfeffer MA, McMurray JJ, Velazquez EJ, Rouleau JL, Kober L, Maggioni AP, et al. Valsartan,

captopril, or both in myocardial infarction complicated by heart failure, left ventricular dysfunction, or both. N Engl J Med. 2003;349(20):1893-906. doi: 10.1056/NEJMoa032292. PubMed PMID: 14610160.
12. Bakris GL, Weir MR. Angiotensin-converting enzyme inhibitor-associated elevations in serum creatinine: is this a cause for concern? Archives of internal medicine. 2000;160(5):685-93. Epub 2000/03/21. doi: 10.1001/archinte.160.5.685. PubMed PMID: 10724055.
13. Brar S, Ye F, James MT, Hemmelgarn B, Klarenbach S, Pannu NJJim. Association of angiotensin-converting enzyme inhibitor or angiotensin receptor blocker use with outcomes after acute kidney injury. 2018;178(12):1681-90.
14. Strauss MH, Hall AS. Angiotensin Receptor Blockers Do Not Reduce Risk of Myocardial Infarction, Cardiovascular Death, or Total Mortality: Further Evidence for the ARB-MI Paradox. Circulation. 2017;135(22):2088-90. doi: 10.1161/CIRCULATIONAHA.117.026112. PubMed PMID: 28559493.
15. Indications for ACE inhibitors in the early treatment of acute myocardial infarction: systematic overview of individual data from 100,000 patients in randomized trials. ACE Inhibitor Myocardial Infarction Collaborative Group. Circulation. 1998;97(22):2202-12. doi: 10.1161/01.cir.97.22.2202. PubMed PMID: 9631869.
16. Bangalore S, Kumar S, Wetterslev J, Messerli FH. Angiotensin receptor blockers and risk of myocardial infarction: meta-analyses and trial sequential analyses of 147 020 patients from randomised trials. BMJ. 2011;342:d2234. doi: 10.1136/bmj.d2234. PubMed PMID: 21521728; PubMed Central PMCID: PMCPMC3082637.
17. Dickstein K, Kjekshus J, Committee OS, Lancet OSGJT. Effects of losartan and captopril on mortality and morbidity in high-risk patients after acute myocardial infarction: the OPTIMAAL randomised trial. 2002;360(9335):752-60.
18. Savarese G, Costanzo P, Cleland JG, Vassallo E, Ruggiero D, Rosano G, et al. A meta-analysis reporting effects of angiotensin-converting enzyme inhibitors and angiotensin receptor blockers in patients without heart failure. J Am Coll Cardiol. 2013;61(2):131-42. doi: 10.1016/j.jacc.2012.10.011. PubMed PMID: 23219304.
19. Bangalore S, Fakheri R, Toklu B, Ogedegbe G, Weintraub H, Messerli FH. Angiotensin-Converting Enzyme Inhibitors or Angiotensin Receptor Blockers in Patients Without Heart Failure? Insights From 254,301 Patients From Randomized Trials. Mayo Clin Proc. 2016;91(1):51-60. doi: 10.1016/j.mayocp.2015.10.019. PubMed PMID: 26763511.
20. Choi IS, Park IB, Lee K, Ahn TH, Kim JH, Ahn Y, et al. Angiotensin-Converting Enzyme Inhibitors Provide Better Long-Term Survival Benefits to Patients With AMI Than Angiotensin II Receptor Blockers After Survival Hospital Discharge. J Cardiovasc Pharmacol Ther. 2018:1074248418795897. doi: 10.1177/1074248418795897. PubMed PMID: 30130974.
21. Kim YH, Her AY, Jeong MH, Kim BK, Hong SJ, Shin DH, et al. Clinical Outcomes at 2 Years Between Beta-Blockade with ACE Inhibitors or ARBs in Patients with AMI Who Underwent

Successful PCI with DES: A Retrospective Analysis of 23,978 Patients in the Korea AMI Registry. Am J Cardiovasc Drugs. 2019;19(4):403-14. doi: 10.1007/s40256-019-00326-8. PubMed PMID: 30788675.
22. Kim YH, Her AY, Jeong MH, Kim BK, Lee SY, Hong SJ, et al. Impact of renin-angiotensin system inhibitors on long-term clinical outcomes in patients with acute myocardial infarction treated with successful percutaneous coronary intervention with drug-eluting stents: Comparison between STEMI and NSTEMI. Atherosclerosis. 2019;280:166-73. doi: 10.1016/j.atherosclerosis.2018.11.030. PubMed PMID: 30529829.
23. Tseng DS, Kwong J, Rezvani F, Coates AO. Angiotensin-converting enzyme-related cough among Chinese-Americans. Am J Med. 2010;123(2):183 e11-5. doi: 10.1016/j.amjmed.2009.06.032. PubMed PMID: 20103031.
24. Evans M, Carrero JJ, Szummer K, Akerblom A, Edfors R, Spaak J, et al. Angiotensin-Converting Enzyme Inhibitors and Angiotensin Receptor Blockers in Myocardial Infarction Patients With Renal Dysfunction. J Am Coll Cardiol. 2016;67(14):1687-97. doi: 10.1016/j.jacc.2016.01.050. PubMed PMID: 27056774.
25. Lin Y-CH, Chiao Ying ; Kao, Chih Chin ; Chen, Tzen-Wen ; Chen, His Hsien ; Hsu, Chi Cheng ; Wu, Mai-Szu. Incidence and Prevalence of ESRD in Taiwan Renal Registry Data System (TWRDS): 2005-2012. Acta Nephrologica. 2014;Vol. 28, No. 2.:pp. 65-8.
26. Fu EL, Evans M, Clase CM, Tomlinson LA, van Diepen M, Dekker FW, et al. Stopping Renin-Angiotensin System Inhibitors in Patients with Advanced CKD and Risk of Adverse Outcomes: A Nationwide Study. J Am Soc Nephrol. 2021;32(2):424-35. doi: 10.1681/ASN.2020050682. PubMed PMID: 33372009; PubMed Central PMCID: PMCPMC8054897.
27. Steiner JF, Prochazka AV. The assessment of refill compliance using pharmacy records: methods, validity, and applications. J Clin Epidemiol. 1997;50(1):105-16. doi: 10.1016/s0895-4356(96)00268-5. PubMed PMID: 9048695.

Figure 1

Click here to access/download;Figure;Fig. 1.tif

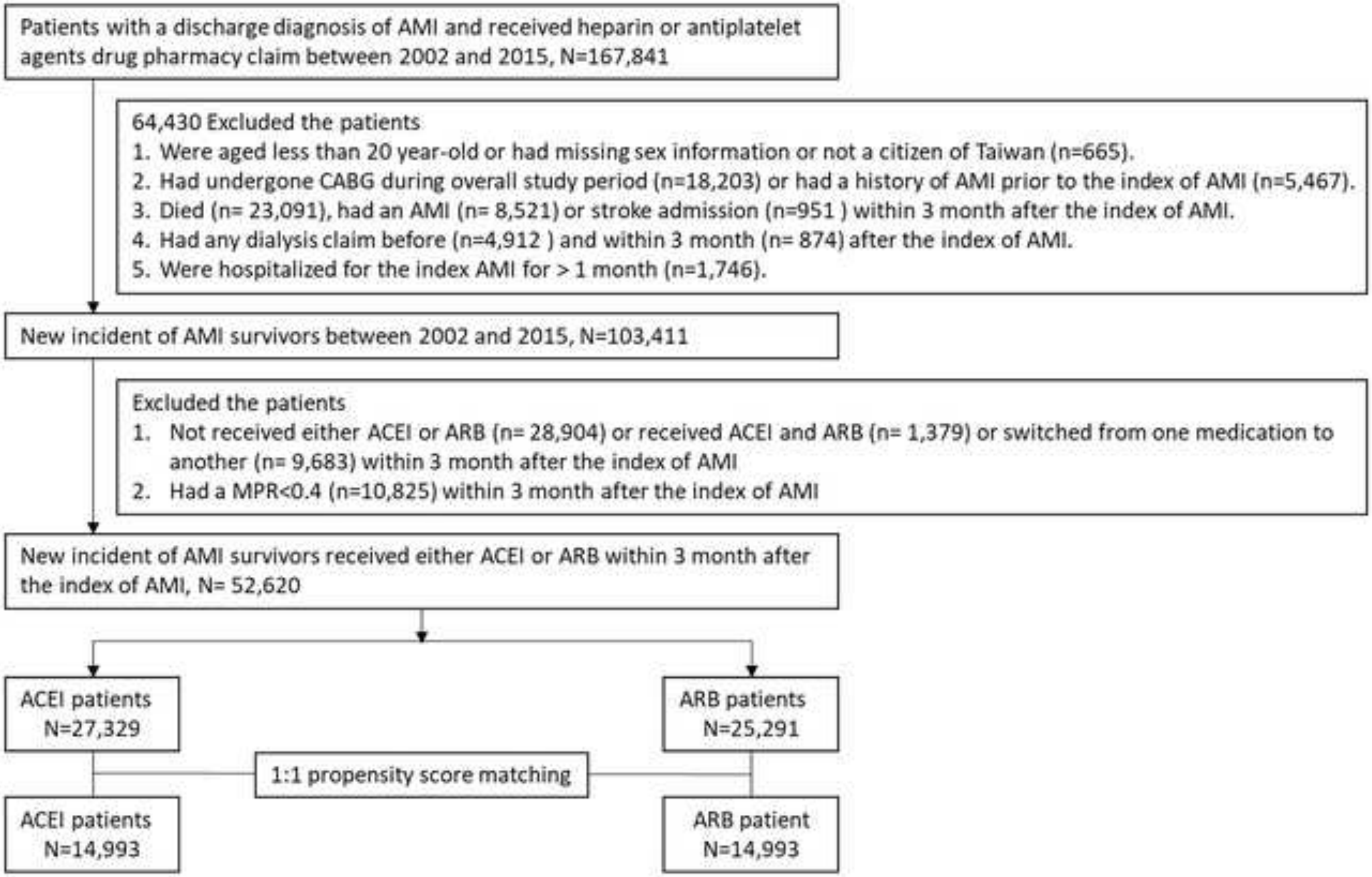

Figure 2a

Click here to access/download;Figure;Fig. 2A.tif

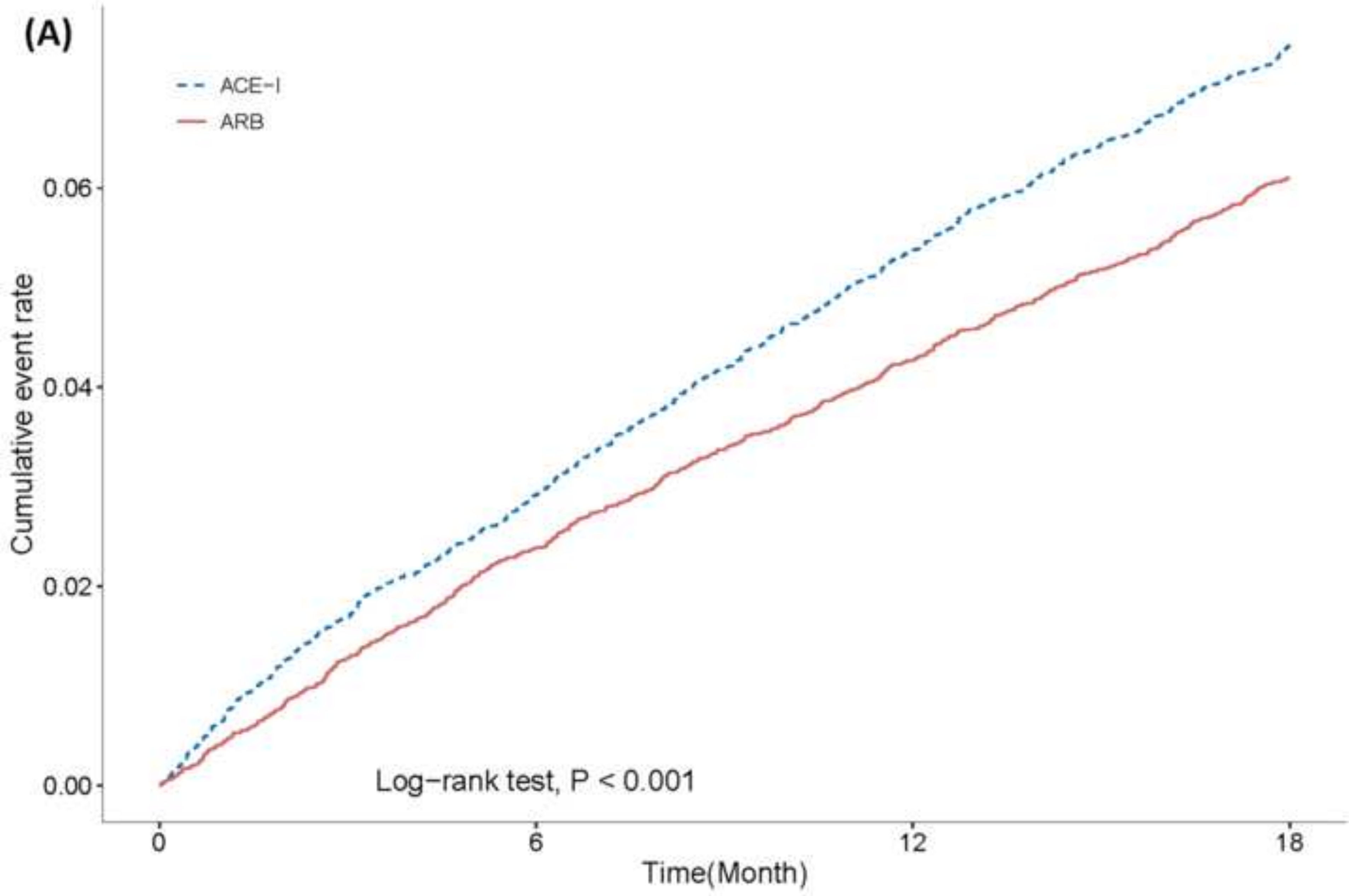

Figure 2b

Click here to access/download;Figure;Fig. 2B.tif

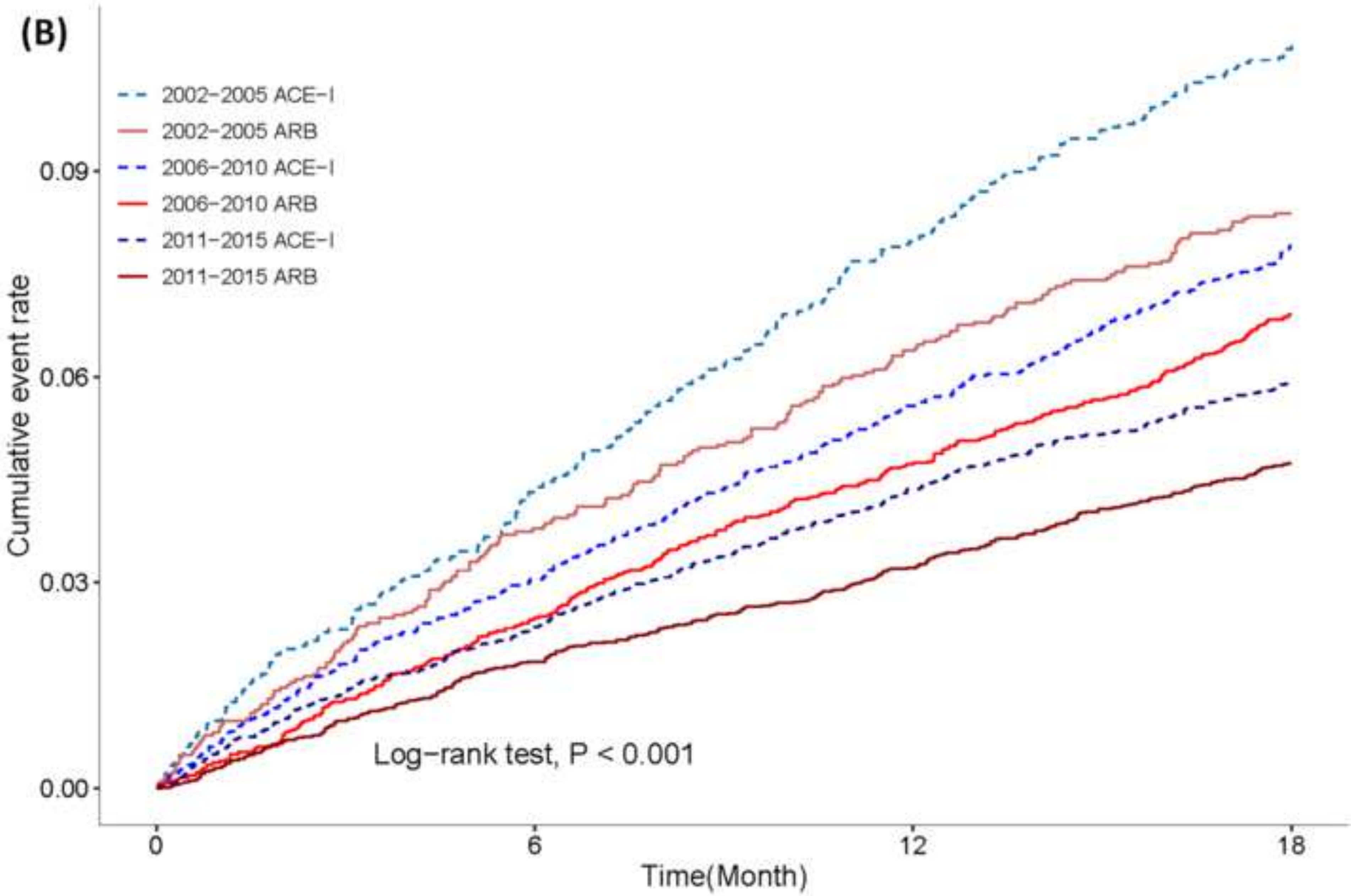

Figure 3

Click here to access/download;Figure;Fig. 3.tif

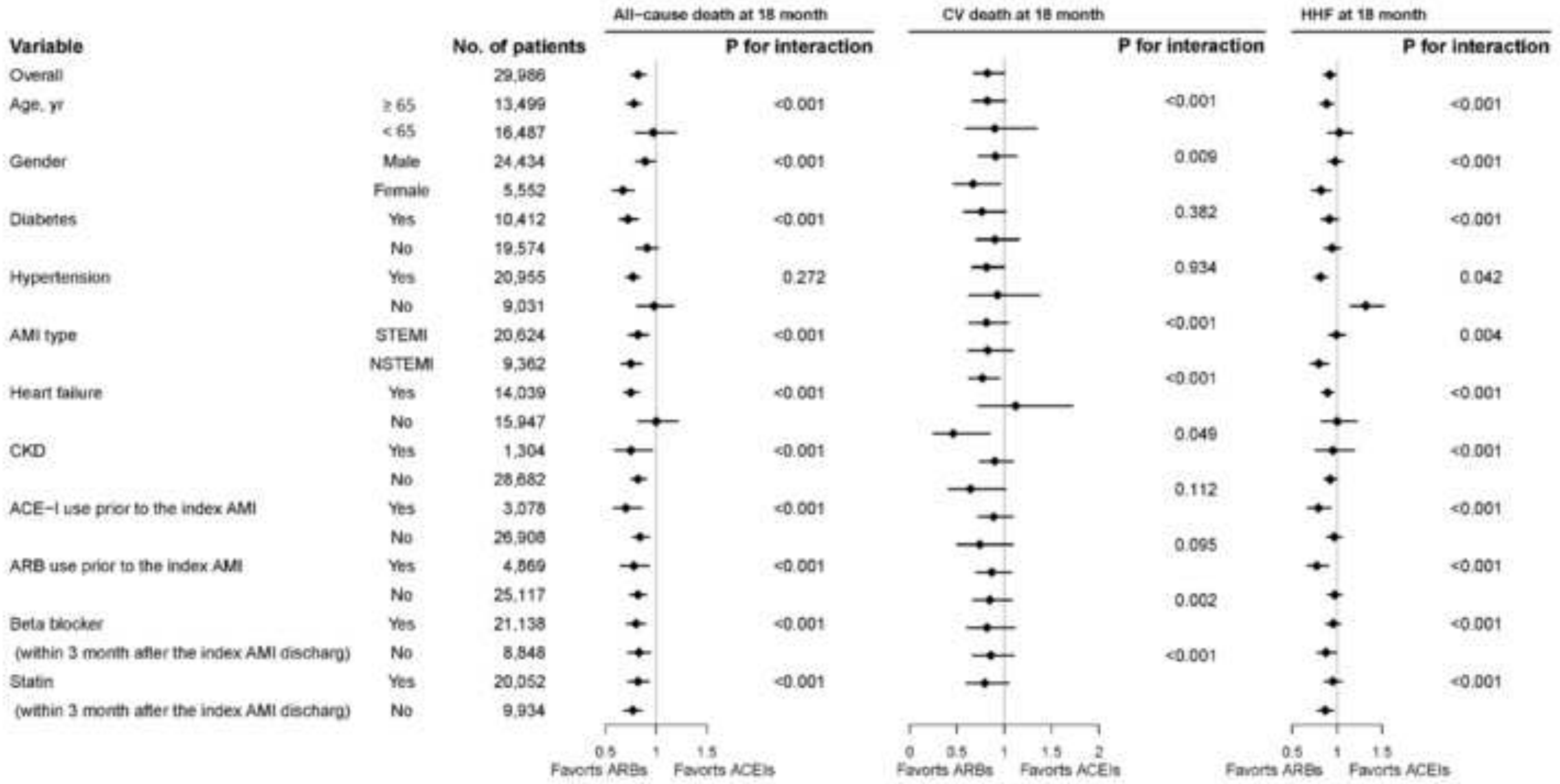

Supplemental material

Click here to access/download
Supporting Information
Supplemental material.docx
