# Supplementary material for: Comparison of clinical outcomes of angiotensin receptor blockers with angiotensin-converting enzyme inhibitors in patients with acute myocardial infarction
Source: PLoS One. 2023 Sep 14;18(9):e0290251. doi: 10.1371/journal.pone.0290251 (PMC10501560; doi:10.1371/journal.pone.0290251)
Supplement: Attachment — Submitted filename: Response to Reviewer 20230601.docx [file pone.0290251.s006.docx]

Dear Editor,

Thank you for considering our manuscript for publication in PLOS ONE. We appreciate the time and effort the academic editor and reviewers invested in evaluating our work. We have carefully reviewed the suggestions, and our responses are as follows. We also revised the manuscript by using track changes where the portion needs to be changed.

For Journal requirements:

1. Please ensure that your manuscript meets PLOS ONE's style requirements, including those for file naming.

Answers:

We revised our manuscript to meet PLOS ONE's style requirements, including the title page, file naming, and author byline.

2. We noticed you have some minor occurrence of overlapping text with the following previous publication(s), which needs to be addressed:- 10.1097/HJH.0000000000000804

In your revision ensure you cite all your sources (including your own works), and quote or rephrase any duplicated text outside the methods section. Further consideration is dependent on these concerns being addressed.

Answers:

Like the referenced article, our study utilized NHIRD to analyze the prognosis of ACEIs/ARBs in different groups (prior stroke and post-myocardial infarction). We also applied similar methods, such as propensity score matching, to reduce potential confounding factors inherent in retrospective data and employed two analysis methods (IIT and as-treated analysis) to address the issue regarding patients' actual medication adherence in the database. Therefore, we referenced the aforementioned article as reference no. 29 and addressed the limitation, as suggested by the reviewers.

Change:

And we revise the description in the limitation section as follows:

(page 19, line 19, discussion paragraph, limitation section) The current study is subject to a few limitations. First, we applied PSM to balance the major observable baseline covariates to reduce the selection bias; however, the residual confounding bias might still exist due to the observational study design. Second, the NHIRD does not provide information on blood pressure, factors associated with drug selection, and dosage of a specific antihypertensive medication. Additionally, patient characteristics such as obesity and smoking status, allergy history, and laboratory data such as potassium levels, blood glucose, successful or unsuccessful PCI, and actual ejection fraction are also not available in the NHIRD. Third, we used the prescription claims to estimate medication adherence which was correlated with pill counts in other studies. Still, actual drug adherence of individuals was unable to obtain. Finally, the interruption or switching of drugs may have resulted in an overestimation of the effects of the respective drugs in the IIT analysis. However, similar findings were found when treating patients who switched their initial medication as censoring, as detailed in S3 and S4 table. Two analysis methods (IIT and as-treated analysis) were used to verify the data from NHIRD, which were also employed in another database study (ref. 29)

3.Please include captions for your Supporting Information files at the end of your manuscript, and update any in-text citations to match accordingly. Please see our Supporting Information guidelines for more information: <http://journals.plos.org/plosone/s/supporting-information>

Answers:

We included table captions for supplemental material at the end of our manuscript. Thank you for your reminder.

Reviewer 1

1. 54% of ACEI users switched to ARBs in a median time of 14 months. And relatively high mortality in the ACEI group compared to ARBs. Is it an ARB effect or other confounders like EF, the unsuccessful PCI, ICD, and door-to-device time?

Answers:

Thank you for the comment. The data of actual left ventricular ejection fraction, successful or unsuccessful PCI, and door-to-device time were unavailable in our NHIRD. Alternatively, we used International Classification of Diseases (ICD) codes to define underlying comorbidities of heart failure and employed propensity score matching to reduce the effect of confounding factors. We addressed the limitation in the discussion.

Change:

(page 20, line 4, discussion paragraph, limitation section) Additionally, patient characteristics such as obesity and smoking status, allergy history, and laboratory data such as potassium levels, blood glucose, successful or unsuccessful PCI, and actual ejection fraction are also not available in the NHIRD.

2. It's better to know the type of stents they use. A random sample from NHID Taiwan (2007- 2010) sample showed disproportionally high usage of BMS(65%) over DES(35%).

<https://journals.plos.org/plosone/article?id=10.1371/journal.pone.0179127>

Answers:

Thank you for the comment. Indeed, due to the reimbursement conditions of the National Health Insurance in Taiwan, the use of bare metal stents (BMS) has been relatively higher in the past (Acta Cardiol Sin 2014;30:553-564), but the utilization rate of drug-eluting stents (DES) has gradually increased in recent years. Our study spanned several time periods (2002-2005, 2006-2010, 2011-2015), and users of angiotensin receptor blockers (ARBs) consistently associated with better all-cause mortality outcomes compared to users of angiotensin-converting enzyme inhibitors (ACEIs) across different time periods. We recognized that we did not consider the information regarding the type of stent; however, the results should not be influenced.

3. Post MI Ejection fraction?
-How many have had improved EF?
-Use imaging during PCI if stent thrombosis is a common cause of mortality?
-How many vessels intervened?
-How many received ICD/CRT?
-Smoking history?

-Door to balloon or device time?

Answers:

Thank you for the comment, as mentioned in the previous point, the data mentioned above were not available in our NHIRD. Therefore, we have modified the description in the limitation section.

Change:

(page 20, line 4, discussion paragraph, limitation section) Additionally, patient characteristics such as obesity and smoking status, allergy history, and laboratory data such as potassium levels, blood glucose, successful or unsuccessful PCI, and actual ejection fraction are also not available in the NHIRD.

4.All-cause mortality is high in the ACEI group. I would like to know the common cause of death in both groups.

Answers:

Thank you for your comment. Our study aims to compare the all-cause mortality and cardiovascular outcomes between users of ACEIs and ARBs in patients with acute myocardial infarction. Thus, we did not report the data on non-cardiovascular death.

5. I wonder how reliable the final results are if we don't have ejection fraction, type of stents, how many and what vessel were revascularized, and success of PCI, primarily if your study population consist of ~65-70% STEMI.

Answers:

Thank you for the suggestions. Indeed, in this retrospective cohort study, we still have several limitations and potential biases, which we have addressed in the limitation section. We, therefore, have employed various methods to mitigate confounding factors in our study, such as using propensity score matching (PSM) and employing different analytical approaches (intention-to-treat and as-treated). Additionally, we utilized the medication possession ratio (MPR) to confirm that our analysis focused on actual medication users of ACEIs or ARBs among AMI patients. The results were also consistent in the sensitivity analyses. We were more confident that this study still holds a certain reference value.

Reviewer 2

1.The discussion explains how is this study different from the previous studies. However, an article has recently been published in PLOS ONE by Jae-Geun Lee titled 'Impact of angiotensin-converting enzyme inhibitors versus angiotensin receptor blockers on clinical outcomes in hypertensive patients with acute myocardial infarction' which also answered this question using national registry in Asian population. Please address how is your study different from this study and what additional information does your study provide besides renal outcomes? This study by Lee JG gives contradictory results and states that ARBs are inferior to ACEI in AMI in Asian population.

Answers:

Thank you for your comment. The reviewer mentioned that the article by Lee JG, similar to other studies using the KAMIR database, analyzes long-term outcomes based on medication usage at discharge for AMI patients. However, medication usage at discharge does not necessarily indicate continued medication usage after discharge. Therefore, we only included the patients whose medication possession ratio (MPR) was > 40% to ensure they had a certain level of ACEIs or ARBs usage following AMI discharge. In the revised manuscript, we have added the article suggested by the reviewer as the 23rd reference in our manuscript and revise the description in the discussion section as follows:

Change: (page 18, line 7, discussion paragraph) Most of these studies were conducted in Western countries and enrolled a few Asians. Furthermore, studies conducted in Asia - most of which used data from the Korea Acute Myocardial Infarction Registry (KAMIR) - have shown inconsistent results [7, 20-23].

(page 19, line 5, discussion paragraph) Our study differs from previous studies in several aspects. Firstly, only a few studies have focused on Asians. Most of these Asian trials used data from KAMIR, which investigated the clinical outcomes of ACEI and ARB users. However, the KAMIR data only evaluated patients' medications at discharge [7, 20, 23]. On the contrary, our study evaluated the actual medication usage within 3 months after discharge of myocardial infarction patients, which can provide a clearer reflection of the clinical outcomes difference for patients who actually used ACEIs and ARBs

2. Patients receiving both ACEI and ARBs were excluded from the study. However, it will be interesting to do a subgroup comparison of patients who received both ACEI and ARBs as compared to ACEI or ARB alone.

Answers:

Thank you for your comment. In our study, the number of patients who concurrently received ACEIs and ARBs was only 1370 individuals (2.6% of the total population). With such a small number, conducting further subgroup analysis may be challenging. On the other hand, findings from the ONTARGET trial (N Engl J Med 2008; 358:1547-1559) suggest that the combination of ACEIs and ARBs may be linked to a higher occurrence of adverse events in patients with cardiovascular risk, without any discernible increase in benefits.

3. In Table 1, under 'Medication Use, yes' sub-heading, ACEI and ARB use is mentioned in both groups. Does this imply that these patients were receiving ACEI and ARB prior to AMI? Yes. If yes, it might be interesting to look into outcomes of patients who were continued on the same medication vs those who started it for the first time after AMI hospitalization.

Answers:

We appreciate your opinion. Yes, in Table 1, the medication use represents the drugs taken by the patients before the index AMI. In fact, in our subgroup analysis depicted in Figure 3, we specifically examined the impact of ACEIs or ARBs usage before the index AMI on mortality outcomes after the index AMI. Based on our analysis results, it appears that there is no significant effect on ARBs users associated with lower mortality than ACEIs users after AMI.

4. Post-hoc analysis of CONSENSUS-II trial has shown that addition of aspirin may reduce ACEI benefit in AMI patients. Even though most of the AMI patients will be receiving aspirin, It will be clinically relevant to look into outcomes of patients on ACEI or ARB with vs without aspirin. Same can be done for beta blockers, CCB, or NOACS if data allows.

Answers:

We appreciate your opinion. In our study population (Table 1), nearly 90% of the patients were using aspirin after AMI (91.8% in ACEIs users, 88.9% in ARBs users). Due to the small proportion of patients not using aspirin, conducting further subgroup analysis may not be appropriate from a statistical perspective. In Figure 3, we performed a subgroup analysis based on using beta-blockers or statins to assess their impact on our outcomes, but the results remained unchanged. The use of NOACs accounted for less than 1% of the analyzed population, and we did not conduct further analysis for CCBs as they are not essential medications after AMI.

5.Some patients might have been prescribed ARBs if ACEI are contraindicated. If it's possible to see if patient was allergic to ACEI or if it was contraindicated, it will be relevant to tabulate outcomes in patients who received ARBs because they were allergic to ACEI vs those who were not allergic.

Answers:

Thank you for your comment. Due to the nature of our study being a retrospective cohort study using data from NHIRD, we did not have access to information regarding allergy history and the factors considered in the selection of ACEIs or ARBs. We would like to add it to the limitation regarding factors associated with medication switching that were unable to obtain in this study.

Change:

(page 20, line 2, discussion paragraph, limitation section) Second, the NHIRD does not provide information on blood pressure, factors associated with drug selection and dosage of a specific antihypertensive medication. Additionally, patient characteristics such as obesity and smoking status, allergy history, and laboratory data such as potassium levels, blood glucose, successful or unsuccessful PCI, and actual ejection fraction are also not available in the NHIRD.

6. A comment should be added at the end whether you conclude that ARBs should be preferred in Asian population or further studies are required to make a recommendation. Studies have shown contradictory results even within Asian population eg. Jae-Geun Lee et al. It will be helpful if a study design is outlined with required variables which will be able to get more definite results.

Answers:

We appreciate your opinion. We have made the following modifications to the "conclusion" section in the discussion

Change:

(page 20, line 16, discussion paragraph, conclusion section) Our study may have clinical implications for Asians after MI. Despite the contradictory results observed in some studies focusing on the Asian population, our analysis only included the patients whose MPR ≥ 40% to ensure the patients were actual medication users of ACEIs or ARBs, suggests that ARBs may be beneficial in reducing all-cause mortality and lowering the hospitalization rate for heart failure compared to ACEIs.

7.ACEI/ARBS have shown to improve LVEF following AMI. It would've been intriguing to study the LVEF improvement but as mentioned in the study exact LVEF data isn't available in the database used. Since, hospitalization for heart failure is one of the main outcomes of the study. It will add to the strength of the study to compare the hospitalizations for heart failure with preserved vs reduced ejection fraction using respective ICD-10 codes.

Answers:

We appreciate your opinion. However, as mentioned earlier, the lack of LVEF data in the NHIRD is a limitation of our study. Using ICD-10 to differentiate hospitalization events for heart failure into preserved or reduced ejection fraction is indeed a good approach. However, since the conversion of NHIRD reporting data from ICD-9 to ICD-10 began in 2016, and our study data spans from 2002 to 2015, hospitalizations for heart failure cannot be obtained with ICD-10 data, making it difficult to distinguish preserved or reduced ejection fraction. Nonetheless, we still appreciate the reviewer for providing us with such valuable research method suggestions.

Sincerely,

We hope that the revised manuscript will meet the expectations of both the reviewers and yourself and await the decision concerning publication of PLOS ONE.

On behalf of all authors,

Li-Nien Chien

Institute of Health and Welfare Policy, College of Medicine, National Yang Ming Chiao Tung University, Taipei, Taiwan

Email address: [linien.chien@nycu.edu.tw](mailto:linien.chien@nycu.edu.tw) (LNC)

Chun-Yao Huang

Division of Cardiology, Department of Internal Medicine and Cardiovascular Research Center, Taipei Medical University Hospital, Taipei, Taiwan

Email address: [cyhuang@h.tmu.edu.tw](mailto:cyhuang@h.tmu.edu.tw) (CYH)
