# Supplementary material for: Comparison of clinical outcomes of angiotensin receptor blockers with angiotensin-converting enzyme inhibitors in patients with acute myocardial infarction
Source: PLoS One. 2023 Sep 14;18(9):e0290251. doi: 10.1371/journal.pone.0290251 (PMC10501560; doi:10.1371/journal.pone.0290251)
Supplement: Attachment — Submitted filename: Response to Reviewer 20230802.docx [file pone.0290251.s007.docx]

Dear Editor,

Thank you once again for your response and consideration to publish our article in the PLOS ONE journal. We truly value the time and effort invested by the academic editor and reviewers in evaluating our work. We have thoroughly reviewed the suggestions and our responses are as follows.

Reviewer 1

I suggest authors to add the following statement and minor revision as follows:

My suggestions:

Conclusion:

I will delete this sentence and put in the discussion section as appropriate if not stated “Our study may have clinical implications for Asians after MI. Despite the contradictory results observed in some studies focusing on the Asian population [7, 20, 23], our analysis only included the patients whose MPR ≥40% to ensure the patients were actual medication users of ACEIs or ARBs”

I will make the conclusion statement simple:

The results of this study suggest that ARBs may be beneficial in reducing all-cause mortality and lowering the hospitalization rate for heart failure compared to ACEIs.

In the conclusion I will add the following statement

Given the retrospective nature with several limitations, this study is hypothesis generating and further studies are needed to evaluate the role of ACEI versus ARB in Asian AMI patients

Answers:

We greatly appreciate your valuable input. Based on the reviewer's suggestion, we have revised our conclusion as follows.

Change:

(page 20, line 14, discussion paragraph, conclusion section) The results of this study suggest that ARBs may be beneficial in reducing all-cause mortality and lowering the hospitalization rate for heart failure compared to ACEIs. Given the retrospective nature with several limitations, this study is hypothesis generating and further studies are needed to evaluate the role of ACEI versus ARB in Asian AMI patients.

We hope that the revised manuscript will meet the expectations of both the reviewers and yourself and await the decision concerning publication of PLOS ONE.

Sincerely,

On behalf of all authors,

Li-Nien Chien

Institute of Health and Welfare Policy, College of Medicine, National Yang Ming Chiao Tung University, Taipei, Taiwan

Email address: [linien.chien@nycu.edu.tw](mailto:linien.chien@nycu.edu.tw) (LNC)

Chun-Yao Huang

Division of Cardiology, Department of Internal Medicine and Cardiovascular Research Center, Taipei Medical University Hospital, Taipei, Taiwan

Email address: [cyhuang@h.tmu.edu.tw](mailto:cyhuang@h.tmu.edu.tw) (CYH)
